# Extrasynaptic α_6_ Subunit-Containing GABA_A_ Receptors Modulate Excitability in Turtle Spinal Motoneurons

**DOI:** 10.1371/journal.pone.0115378

**Published:** 2014-12-22

**Authors:** Carmen Andres, Justo Aguilar, Ricardo González-Ramírez, David Elias-Viñas, Ricardo Felix, Rodolfo Delgado-Lezama

**Affiliations:** 1 Departamento de Fisiología, Biofísica y Neurociencias, Centro de Investigación y Estudios Avanzados del Instituto Politécnico Nacional (Cinvestav-IPN), México DF, México; 2 Departamento de Biología Molecular e Histocompatibilidad, Hospital General “Dr. Manuel Gea González,” México DF, México; 3 Departamento de Bioelectrónica, Cinvestav-IPN, México DF, México; 4 Departamento de Biología Celular, Cinvestav-IPN, México DF, México; Inserm, France

## Abstract

Motoneurons are furnished with a vast repertoire of ionotropic and metabotropic receptors as well as ion channels responsible for maintaining the resting membrane potential and involved in the regulation of the mechanisms underlying its membrane excitability and firing properties. Among them, the GABA_A_ receptors, which respond to GABA binding by allowing the flow of Cl^−^ ions across the membrane, mediate two distinct forms of inhibition in the mature nervous system, phasic and tonic, upon activation of synaptic or extrasynaptic receptors, respectively. In a previous work we showed that furosemide facilitates the monosynaptic reflex without affecting the dorsal root potential. Our data also revealed a tonic inhibition mediated by GABA_A_ receptors activated in motoneurons by ambient GABA. These data suggested that the high affinity GABA_A_ extrasynaptic receptors may have an important role in motor control, though the molecular nature of these receptors was not determined. By combining electrophysiological, immunofluorescence and molecular biology techniques with pharmacological tools here we show that GABA_A_ receptors containing the α_6_ subunit are expressed in adult turtle spinal motoneurons and can function as extrasynaptic receptors responsible for tonic inhibition. These results expand our understanding of the role of GABA_A_ receptors in motoneuron tonic inhibition.

## Introduction

The γ-aminobutiric acid (GABA) is the major inhibitory neurotransmitter in the mature central nervous system. By activating on specific receptors, GABA inhibits neuronal excitability [1]–[3]. There are two main classes of GABA receptors: GABA_A_ and GABA_B_. The GABA_A_ receptor is ionotropic and consists of a pentameric protein complex which, in addition to binding sites for GABA, involves binding sites for benzodiazepines, barbiturates and other drugs such as furosemide. In an open state this receptor is preferentially permeable to Cl^−^ ions. The binding of two molecules of GABA induces its opening and the influx of Cl^−^ ions causes membrane hyperpolarization [4]. The GABA_B_ receptors, present in the form of dimers, are metabotropic receptors coupled to G proteins [5].

Two subtypes of GABA_A_ receptors have been described in neurons from the hippocampus and cerebellum: synaptic and extrasynaptic. These receptors can be discriminated by their location and subunit composition, as well as by their pharmacological and biophysical properties [1]–[3]. Synaptic receptors mediate fast inhibition while extrasynaptic receptors produce a tonic inhibition. Our knowledge regarding the molecular composition of the GABA_A_ receptor has increased considerably over recent years. At least, six α, three β, one δ, and three γ subunits have been identified in mammals. This molecular diversity greatly contributes to the functional and pharmacological heterogeneity of the GABA_A_ receptors [6].

Synaptic receptors are mainly composed of α_1_–α_3_ subunits, while extrasynaptic contain predominantly α_4_–α_6_ [1]–[3]. Both subtypes of GABA_A_ receptors are blocked by picrotoxin, bicuculline and gabazine [1]. Interestingly, the antagonist furosemide selectively blocks α_6_ subunit-containing extrasynaptic GABA_A_ receptors [1], [3], [7]–[9].

The expression of GABA_A_ receptors with different subunit composition has been evidenced in spinal cord using *in situ* hybridization, RT-PCR and immunofluorescence, and in motoneurons it has been suggested the presence of GABA_A_ receptors containing α_1_–α_5_ subunits [10]–[13]. We have previously shown that extrasynaptic GABA_A_ receptors expressed in motoneurons and primary afferents are tonically activated by ambient GABA, and that the activation of these receptors may modulate the monosynaptic reflex (MSR) [14]–[17]. In addition, we found that blockade of GABA_A_ receptors with low concentrations (1–20 µM) of picrotoxin and gabazine reverted presynaptic inhibition of primary afferents without facilitating the MSR, but depressing the dorsal root potential (DRP). However, when picrotoxin concentration was increased to 100 µM, the MSR was facilitated producing a long lasting activation of some motoneurons accompanied with an additional depression of the DRP [15].

Likewise, we have shown that motoneurons exhibit a GABAergic tonic inhibitory current activated by ambient GABA, though the identity of the α subunit(s) in these receptors is presently unknown [16]. Hence, the main aim of this study was to investigate whether furosemide-sensitive α_6_ subunit-containing GABA_A_ receptors are expressed in motoneurons and mediate tonic inhibition. Our results indicate that furosemide increases the excitability and shifted the holding current of voltage clamped motoneurons. Moreover, molecular biology and biochemical assays using specific probes and antibodies revealed the expression of the α_6_ subunit in motoneurons of the adult turtle spinal cord.

## Materials and Methods

### Preparation

Forty adult turtles (*Trachemys scripta spp*, 15–20 cm carapace length) were anaesthetized with pentobarbitone (100 mg/kg, IP). The plastron was opened and the blood removed by intraventricular perfusion with Ringer solution (∼10°C) of the following composition (in mM): 120 NaCl, 5 KCl, 15 NaHCO_3_, 3 CaCl_2_, 2 MgCl_2_ and 20 glucose saturated with 2% CO_2_ and 98% O_2_ to attain pH 7.6. The lumbar spinal enlargement was isolated by a laminectomy and cut transversally to obtain segments of 2–3 mm and slices of 200–300 µm thick. For electrophysiological recording, the spinal cord segments were placed in a recording chamber and superfused with Ringer solution (20–22°C). At the end of the dissection the animals were killed by decapitation. All experimental procedures followed the guidelines set out in the Journal of Physiology for ethical matters [18] and were carried out with the approval of the Cinvestav-IPN Experimental Ethics Committee and in accordance with the current Mexican Norm for Care and Use of Animals for Scientific Purposes. The animals were provided by the National Mexican Turtle Centre located in Mazunte, Oaxaca, with the authorization (DGVS-03821/0907) of the Ministry of Environment and Natural Resources (Semarnat, Mexico).

### Electrophysiology

Motoneurons were recorded intracellularly with sharp electrodes (20–40 MΩ) filled with potassium acetate (0.8 M) and KCl (0.2 M). Cells were classified as motoneurons if their input resistance was <80 MΩ, presented action potentials (APs) with fast and slow posthyperpolarization and showed adaptation during AP firing [14], [19]. The presence of the GABAergic tonic current was determined in motoneurons by using the visualized patch clamp technique in its whole-cell configuration. The patch pipettes (resistance of 5–10 MΩ) were made from thick-walled borosilicate glass capillaries using a programmable horizontal micropipette puller (Sutter Instruments, Novato, CA) and were filled with the following solution (in mM): 122 CsCl; 5 Na_2_-ATP; 2.5 MgCl_2_; 0.0003 CaCl_2_; 5.6 Mg-gluconate; 5 K-Hepes; 5 Hepes. Motoneurons were identified within the ventral horn of the spinal cord by its large size with the aid of an upright microscope using oblique illumination and the recordings were performed by using the MultiClamp-700B amplifier (Molecular Devices, Union City, CA). The maximal acceptable series resistance compensation was 20%. As described earlier, these cells presented APs with fast and slow posthyperpolarization and adaptation in their firing patterns. Recorded signals were digitized at 20 KHz, filtered using the 8-pole Bessel (2 KHz) and stored in the hard disk of a personal computer for off-line analysis.

### Reverse transcription-polymerase chain reaction (RT-PCR)

Total RNA was extracted from the lumbar enlargement of the spinal cord using TRIzol reagent according to the manufacturer's instructions (Invitrogen, Carlsbad, CA). For cDNA synthesis, total RNA samples (5 µg) were subjected to reverse transcription with 3 µl random primers (50 ng/µl) and 1 µl (200 U) M-MLV RT enzyme (Invitrogen) in 20 µl of reaction mixture at 37°C for 50 min. cDNA amplification was carried out by PCR in a total volume of 50 µl: 5 µl of cDNA, 1× PCR buffer (20 mM Tris-HCl, 50 mM KCl, pH 8.4), 0.2 mM of each deoxynucleotide triphosphate, 1.5 mM MgCl_2_, 0.5 µM of each primer and 2.5 U of Taq DNA polymerase (Invitrogen) on a PCR thermal cycler (Thermo Fisher Scientific). PCR primers were designed to amplify conserved regions of several species including chicken (*Gallus gallus*), zebrafish (*Danio rerio*), mouse (*Mus musculus*), rat (*Rattus novergicus*) and human (*Homo sapiens*). For the α_6_ subunit, the forward primer sequence was 5′-TATACGTGGAAAAAAGGACC-3′ and the reverse primer sequence was 5′-CTGATGCTCAAAGTGGTCAT-3′; while for actin, the forward primer sequence was 5′-AAGATGACCCAGATCATGTT-3′ and the reverse primer sequence was 5′-GAGTACTTGCGCTCAGGAGG-3′. The PCR reaction was performed as follows: 30 cycles of 95°C for 45 s, 55°C for 30 s and 72°C for 1 min. PCR products were electrophoresed on 1% agarose gels, stained with ethidium bromide and analyzed under ultraviolet light. The identity of the amplicons was confirmed by automated sequencing.

### Western blot

The lumbar enlargement of the adult turtle spinal cord was homogenized in lysis buffer containing 50 mM Tris-HCl pH 8.0, 150 mM NaCl, 0.5 mM phenylmethylsulfonyl fluoride (PMSF), 1% NP-40 and Complete 1× (Roche). The resulting lysates were centrifuged at 12,000×g for 2 min to remove cellular debris. Protein concentration was determined by the bicinchoninic acid method. One hundred µg of proteins were mixed with Laemmli sample buffer and boiled for 5 min. Proteins were separated on 10% SDS-PAGE and transferred onto nitrocelulose membranes (Biorad). Membranes were blocked for 2 h at room temperature in TBS-T (150 mM NaCl, 10 mM Tris-HCl, pH 8, 0.05% Tween 20) containing 8% low-fat dried milk and then incubated overnight at 4°C with the anti-α_6_ subunit antibody (Sigma-Aldrich, St. Louis, MO). After three washes in TBS-T, membranes were incubated with a horseradish peroxidase conjugated secondary antibody (Jackson ImmunoResearch, West Grove, PA). Protein bands were detected using an enhanced chemiluminescence system (Millipore, Bedford, MA).

### Spinal cord immunostaining

Spinal cord sections of 30 µm were first incubated with an anti-ChAT primary antibody (24 h at 4°C, 1∶50, Millipore) and then revealed using a FITC donkey anti-goat secondary antibody (2 h at room temperature, 1∶200, Jackson ImmunoResearch). Subsequently, sections were incubated with an anti-α_6_ subunit antibody (2 h at 4°C, Sigma; 1∶50 dilution), and then exposed 1 h to the secondary antibody (1∶200; Dylight-Jackson donkey; Jackson ImmunoResearch). Samples were examined using confocal laser scanning microscopy (Leica TCS SP2). Images were obtained with the filter set for Dylight 549 using the 20x and the 40x oil immersion plan apochromatic objective (NA 0.8).

### Drugs

GABA_A_ receptors were activated with muscimol (5 µM) or GABA (10–60 µM) and blocked with picrotoxin (20–100 µM) and furosemide (200 µM) applied to the bath solution. Ionotropic glutamatergic and glysinergic receptors were blocked with 6-Cyano-7-nitroquinoxaline-2,3-dione (CNQX; 20 µM), and (2R)-amino-5-phosphonovaleric acid (APV; 40 µM) and strychnine (2 µM), respectively. All drugs were purchased from Sigma-Aldrich (St. Louis, MO).

### Data analysis

The effect of activation and blockade of GABA_A_ receptors in motoneurons recorded intracellularly were quantified by measuring the input resistance and excitability before and after drug application. The input resistance was determined by linear regression through the current-voltage relationship. The excitability was evaluated by plotting the intensity of the current *versus* the number of action potentials produced by supra-threshold intracellular depolarizing current pulses. A change in excitability was indicated by a left shift in the resulting curve.

The mean holding current recorded in voltage clamp experiments was calculated by generating all-point histograms of the current values recorded for 5 s in control Ringer and in the presence of the GABA_A_ receptor blocker. A Gaussian distribution was fitted to the histograms. Changes in holding current values were determined as the difference between the means of the Gaussians fitted to the histograms. The statistical differences between means were determined by Student *t*-tests and Kolmogorov-Smirnov tests. Means were considered statistically different when p<0.05. Values are presented as the mean ± S.E.

## Results

### Identification of motoneurons recorded intracellularly

**Figure 1:**
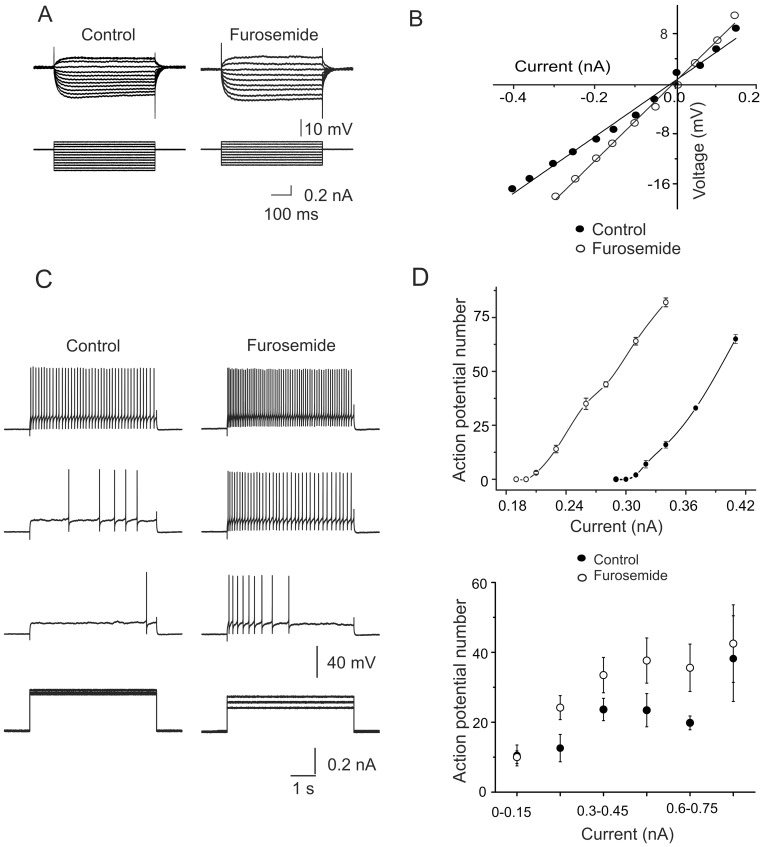
Furosemide-sensitive GABA_A_ receptors modulate motoneuron excitability. A) Current-voltage responses recorded intracellularly from one motoneuron in control Ringer and in the presence of furosemide (200 µM). B) Current-voltage plot from responses obtained as in A. Input resistance was 39 and 83 MΩ in control Ringer and in the presence of furosemide, respectively. C) Action potential firing at different depolarizing intracellular current pulses recorded intracellularly in control Ringer and in the presence of furosemide (200 µM). D) Plot of the depolarizing current pulses *versus* the number of APs evoked in one cell (upper panel) and a group of cells (lower panel) in control Ringer and in the presence of furosemide.

The function of GABA_A_ receptors was studied in 32 motoneurons recorded intracellularly in the bridge mode from adult turtle thick spinal cord slices (2–3 mm). Cells included in this study presented an input resistance of ∼25±4 MΩ, APs with the typical fast and slow posthyperpolarization and adaptation of the firing pattern produced by a long intracellular depolarizing current pulse (Fig. 1A and 1B). These parameters are similar to those reported previously for mature motoneurons [14], [19].

### Furosemide increases motoneuron excitability

**Figure 2:**
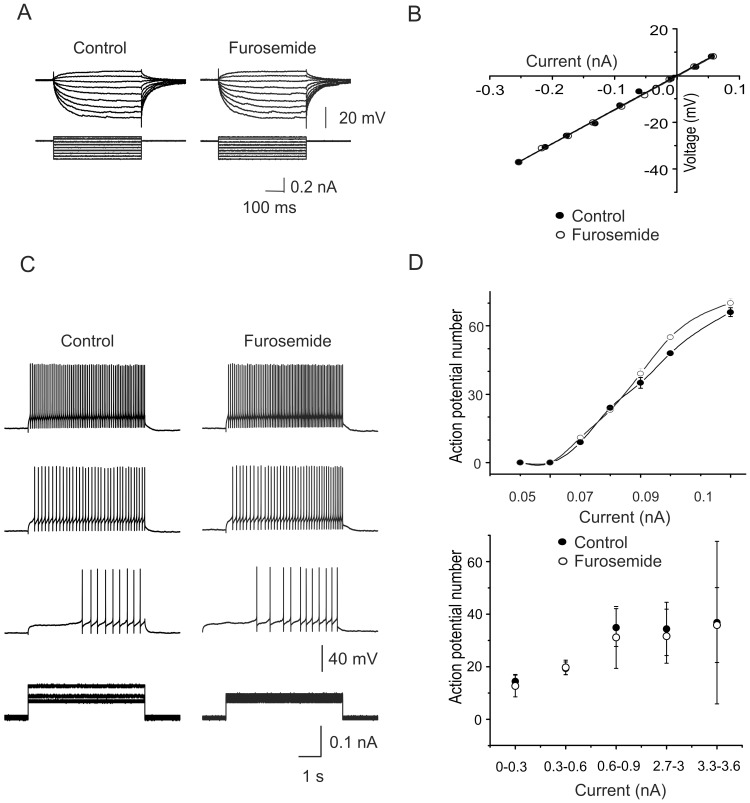
Furosemide non-sensitive GABA_A_ receptors. A) Current-voltage responses recorded intracellularly from one motoneuron in control Ringer and in the presence of furosemide (200 µM). B) I-V curves obtained as in A. C) AP firing in response to different depolarizing intracellular current pulses recorded in control Ringer and in the presence of furosemide (200 µM). D) Plot of the depolarizing current pulses *versus* the number of action potentials evoked in one cell (upper panel) and a group of cells (lower panel) in control Ringer and in the presence of furosemide. All the recordings were obtained in the presence of strychnine (2 µM), CNQX (20 µM) and APV (20 µM).

Previously, we reported that the monosynaptic reflex evoked by electrical stimulation of one dorsal root in the adult turtle spinal cord was facilitated by furosemide without affecting the dorsal root potential [15]. Knowing that furosemide increases the excitability of cerebellar granule cells by blocking extrasynaptic α_6_ subunit-containing GABA_A_ receptors [8], [9], [20], [21], we decided to test whether the facilitation of the monosynaptic reflex was mediated by a similar mechanism in motoneurons. Fig. 1A and 1B show the voltage responses and the resulting *I-V* plots, respectively, evoked by intracellular depolarizing current pulses in a motoneuron in the control condition and in the presence of furosemide (200 µM). In this cell, furosemide application increased the input resistance from about 43 to 64 MΩ and resulted also in increased AP firing compared to the control (Fig. 1C). The latter effect can be clearly visualized in the excitability plots shown in the upper panel of Fig. 1D, where the control curves for an individual cell (upper panel) and a group of cells (*n* = 19; lower panel) are notably shifted to the left in the presence of the drug (in Fig. 1D). In average, furosemide increased the input resistance (R_in_) in ∼49±10% (*n* = 20; p<0.05). These results suggest the expression of α_6_GABA_A_ receptors in motoneurons. It should be noted, however, that in 12 out of 32 motoneurons furosemide did not affect the R_in_ or the excitability curve (Fig. 2), and that no relationship between R_in_ and furosemide sensitivity was observed.

### Electrophysiological recording of the GABAergic tonic inhibitory current in motoneurons

**Figure 3:**
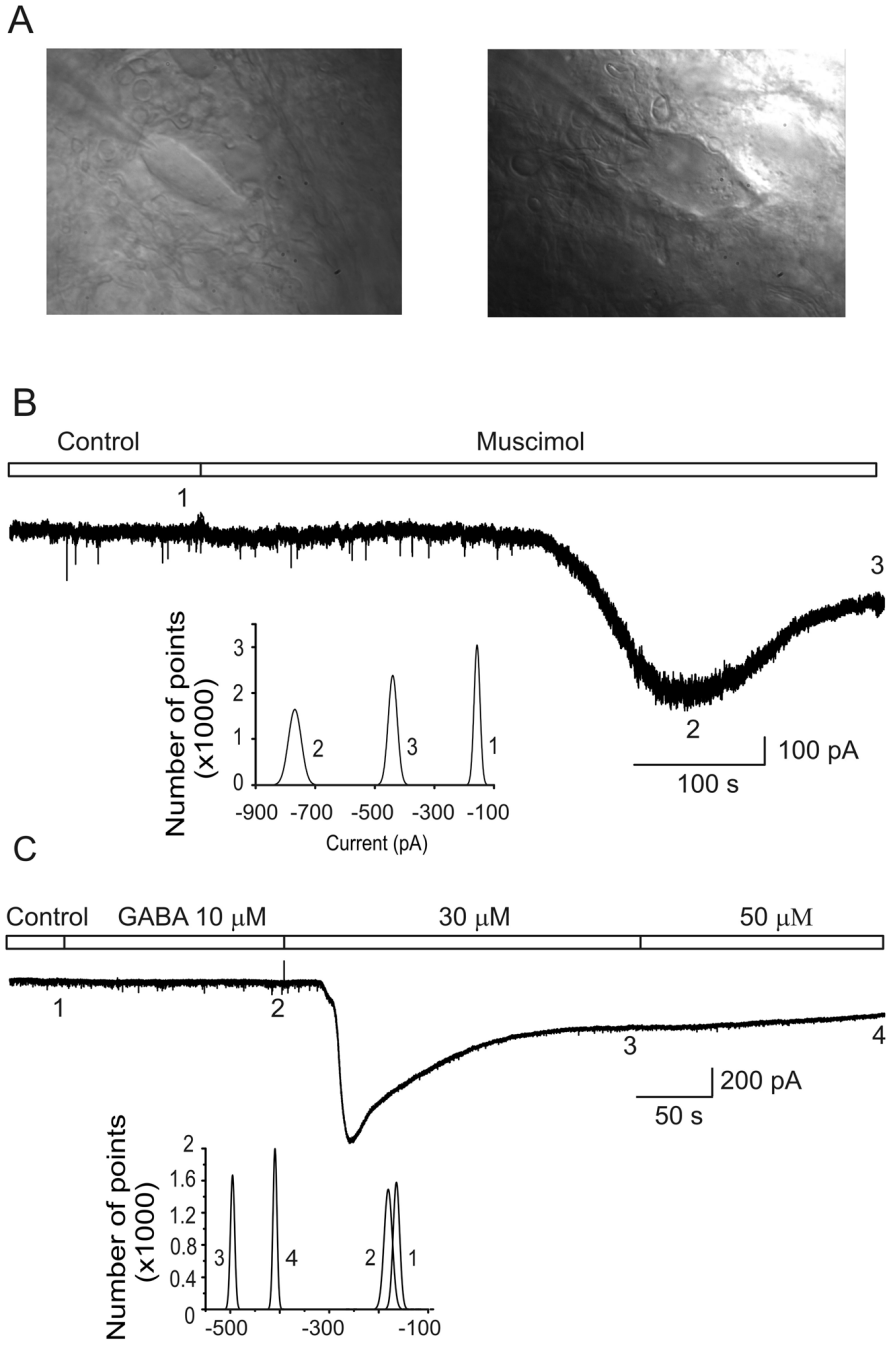
Recording of a GABAergic tonic inhibitory current in motoneurons. A) Images of two patched motoneurons from the ventral horn of the turtle spinal cord viewed with an upright microscope using oblique illumination. B) Representative trace of the holding current recorded in one motoneuron held at −70 mV in control Ringer and in the presence of the GABA_A_ receptor agonist muscimol (5 µM). C) Trace recording of the holding current recorded from one motoneuron held at −70 mV before (control Ringer) and after application of increasing GABA concentrations as indicated. The numbers under the trace denote the time at which 5000 current points were taken to build the histograms shown in the inset.

We have also shown that motoneurons display a GABAergic inhibitory tonic current mediated by GABA_A_ receptors activated by ambient GABA, though the type of α subunit(s) in these receptors was not investigated [16]. Given that furosemide increased the excitability of motoneurons (Fig. 1) and that cerebellar granule cells express a furosemide-sensitive GABAergic inhibitory tonic current mediated by extrasynaptic α_6_ subunit-containing GABA_A_ receptors [1], [8], [9], [21], we next investigated whether the tonic GABAergic current recorded in motoneuron was sensitive to furosemide. By visualizing the ventral horn of the lumbar spinal cord, motoneurons were identified by their location and size and were selected for patch clamp recordings (Fig. 3A). After obtaining the gigaseal and breaking into the cell, recordings were made in the current clamp mode to test the distinctive properties of the motoneurons, such as the adaptation of tonic firing and the presence of the fast and slow posthyperpolarization of APs. Then, by switching to the voltage clamp mode, we investigated the presence of a GABAergic tonic inhibitory current by activating GABA_A_ receptors with muscimol (5 µM).

Fig. 3B shows the holding current recorded in one motoneuron held at −70 mV in the presence of strychnine (2 µM), CNQX (20 µM) and APV (40 µM) after muscimol application (5 µM). As can be seen, muscimol activated a transient inward current with maximal amplitude of ∼650 pA that desensitized to a steady-level of ∼200 pA. This response was associated with an increase in current noise (Fig. 3B). Interestingly, the persistent current which might be produced by the slow desensitizing of some GABA_A_ receptors, had an average amplitude of −397±107 pA in 9 motoneurons treated with muscimol, two-fold larger in comparison with the cells in the control condition (p<0.05). Likewise, the input resistance in cells treated with muscimol was decreased in 51±7% (p<0.05). A Similar reduction in motoneuron R_in_ in the presence of muscimol has been previously reported [14], [16].

In order to facilitate the characterization of the GABA_A_ receptors, we first searched for the optimum exogenous GABA concentration needed to activate the tonic current in our preparation. Fig. 3C shows the holding current recorded in one motoneuron at increasing concentrations of GABA. As can be seen, at concentrations below 10 µM the holding current was not affected, however, concentrations of GABA above 30 µM evoked a fast inward current with an average amplitude of −577±124 pA (*n* = 7), that was followed by a slower desensitization phase that reached a steady state level of −308±41 pA. This value was statistically different to the control (−264±40 pA; p<0.05; *n* = 7). In the presence of GABA, synaptic activity was abrogated most likely due to the shunt produced by the massive activation of GABA_A_ receptors. Likewise, AP firing was activated due to a strong depolarization induced by the efflux of Cl^−^ ions because in these experiments the potential chloride equilibrium potential (E_Cl_−) was close to 0 mV. Therefore, we next decided to investigate the actions of furosemide on the holding current in the presence of 40 µM of GABA. This concentration is close to the EC_50_ for the neurotransmitter (∼45 µM) reported previously for cerebellar granule cells [22].

### GABA_A_ receptors containing the α_6_ subunit mediate tonic inhibition in motoneurons

**Figure 4:**
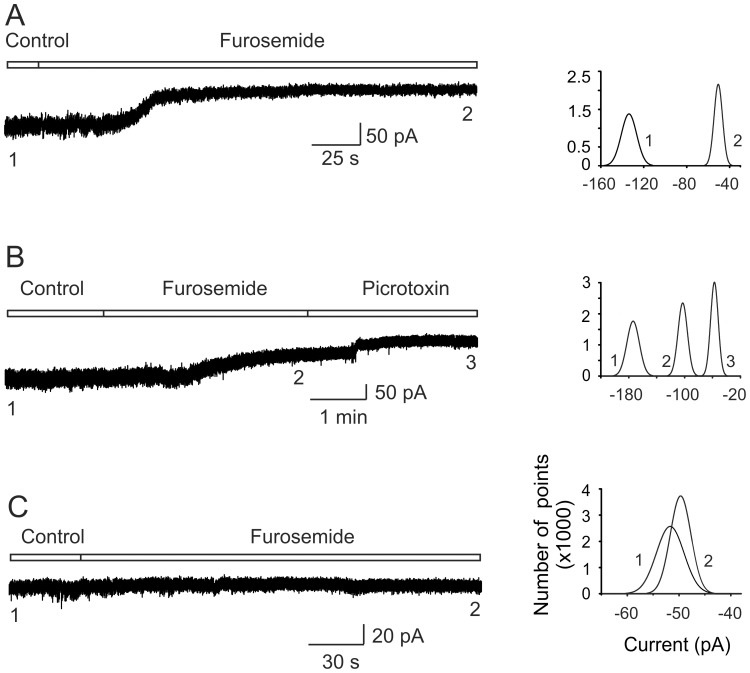
Furosemide sensitive GABAergic tonic current in motoneurons. A) Representative trace of the holding current recorded in one motoneuron held at −70 mV in the presence of furosemide. B) Trace recording of the holding current in another motoneuron in the presence of furosemide after sequential application of picrotoxin. C) Representative trace of the holding current recorded in one motoneuron held at −70 mV in the presence of furosemide. Note that the cell did not respond to drug application. To the right are the histograms built with 500 points taken from the traces at the times indicated by the numbers under the current traces. Recordings in A and B were taken in presence of GABA (40 µM), strychnine (2 µM), CNQX (20 µM) and APV (20 µM). In C, the recordings were made without GABA addition.

Unless otherwise indicated, all motoneurons were recorded in the presence of GABA (40 µM) in combination with a cocktail containing (in µM) 2 strychnine, 20 CNQX and 20 APV antagonists of glycine, AMPA-kynate and NMDA receptors, respectively. As shown earlier, furosemide (200 µM) increased motoneuron excitability (Fig. 1), therefore we next decided to investigate whether this action was due to the blockade of a GABAergic tonic inhibitory current in cells held at −70 mV. Fig. 4A, shows the holding current recorded from a motoneuron in control Ringer and the change produced (83±14 pA) in the presence of furosemide (200 µM). In average, the drug evoked an outward current of 94±36 pA (control, −256±50 pA; furosemide, −162±27 pA; p<0.05, *n* = 22) and an increase in R_in_ of 64±29% (control, 303±155 MΩ; furosemide, 408±167 MΩ; p<0.05, *n* = 5), similar to what was observed in motoneurons recorded intracellularly (Fig. 1).

Likewise, it has been shown that in some neurons the GABAergic tonic current is mediated by more than one type α subunit [1], [3], therefore we asked whether this is occurring also in motoneurons by applying picrotoxin (100 µM) once furosemide action reached steady-state. Fig. 4B shows that picrotoxin produced an additional outward current of 69±22 pA (furosemide, −163±28 pA; picrotoxin: −94±13 pA; p<0.05, *n* = 18), suggesting the expression of another α subunit in addition to α_6_ in motoneurons. Last, it is worth mentioning that in 10 out of 37 motoneurons furosemide did not significantly affected the holding current (Fig. 4C) and did not produce any changes in R_in_, as occurred also in some motoneurons recorded intracellularly (Fig. 2). This insensitivity to furosemide might be associated to differential expression of α_6_GABA_A_ receptors in motoneurons.

### α_6_GABA_A_ receptor expression in the spinal cord of the adult turtle

**Figure 5:**
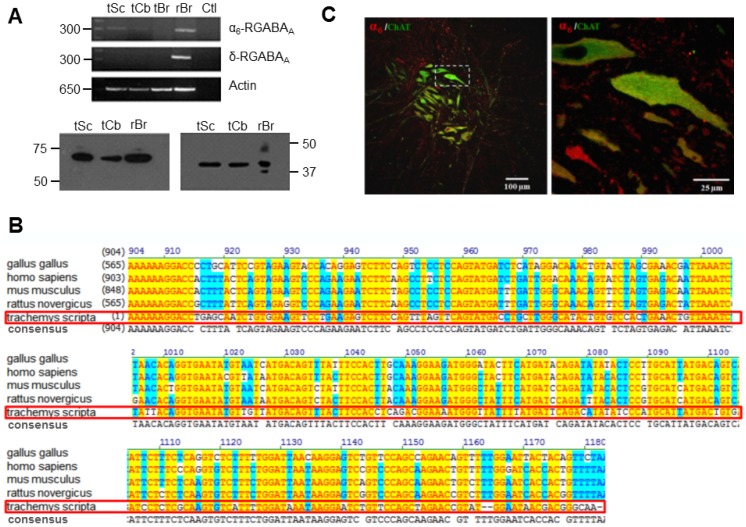
Expression of the α_6_ subunit of the GABA_A_ receptor in the adult turtle spinal cord. A) RNA was extracted from the adult turtle spinal cord (tSc), cerebellum (tCb) and rat brain (rBr) used as a positive control, and subjected to RT-PCR with specific primers for the α_6_ and δ GABA_A_ receptor subunits. Samples containing RNA which was not reverse transcribed were included as negative controls (Ctl). In the bottom panel actin amplification is shown as loading control. B) Proteins extracted from the turtle spinal cord (tSc) cerebellum (tCb) and rat brain (rBr; used as a positive control) were subjected to Western-blot using anti-α_6_ antibodies. A ∼57 KDa band corresponding to the molecular mass of the α_6_ subunit was present in the tSc and tCb lanes as well as in the rBr (left panel). In the right panel a membrane probed with anti-actin antibody is shown as loading control. C) The left panel shows a representative confocal micrograph from an adult turtle spinal cord slice immunostained with choline acetyltransferase (ChaT; a marker for motoneurons) shown in green and with anti-α_6_ antibodies shown in red, suggesting co-localization of both proteins (orange/yellow). Scale bar  = 100 µm. The right panel shows an enlargement of the boxed region in the left panel. Scale bar  = 25 µm.

Previous studies using *in situ* hybridization have shown the expression of RNA for α_2_, α_4_ and α_5_ GABA_A_ receptor subunits in rat and mouse motoneurons [10], [13], [23], [24], though the expression of α_6_ has not been reported yet. Therefore, we next sought to determine whether this subunit is expressed in the spinal cord of the adult turtle. To this end, specific primers directed toward conserved regions in the α_6_ subunit sequences were designed, and total RNA samples from the spinal cord were analyzed by RT-PCR. The results of this analysis showed the presence of a band of the expected size (300 bp) corresponding to the α_6_ subunit (Fig. 5A). The identity of the α_6_ amplicon was confirmed by comparison to the positive control obtained from turtle cerebellum and rat brain RNA samples and by automated sequencing (Fig. 5B). Conventional multiple sequence alignment of the turtle spinal cord α_6_ subunit revealed 97.5% overall identity within different species (Fig. 5B). The sequence reported in this paper is also being deposited in the GenBank database. Last, given that the δ subunit has been also associated to extrasynaptic GABA_A_ receptors we investigated its expression, however, no amplification was observed in the turtle spinal cord (Fig. 5A).

The second line of experimental evidence supporting the expression of α_6_ subunit in the adult turtle spinal cord was obtained using antibodies. Western blot analyses of rat brain, as well as cerebellum and adult turtle spinal cord homogenates with α_6_ subunit antibodies showed a prominent band (Fig. 5C) of the expected mass for the full-length α_6_ protein (∼57 kDa). Last, to determine whether the α_6_ subunit is expressed specifically in motoneurons, immunohistochemical staining was performed on transverse slices of the turtle lumbar spinal cord. The results of this analysis show that the α_6_ immunostaining is prominent in cells co-expressing choline acetyltransferase (a marker for motoneurons), where signal was dispersedly distributed in the soma and proximal dendrites, sparing the nucleus (Fig. 5D). Interestingly, in some motoneurons the signal was very low likely corresponding to furosemide-insensitive cells. No labeling was seen in the absence of the primary antibody or in presence of its corresponding antigenic peptide.

## Discussion

In this study we show that motoneurons recorded intracellularly and in the whole-cell mode of the patch clamp technique express furosemide-sensitive GABA_A_ receptors which modulate their excitability and mediate a tonic inhibitory current. These results are in agreement with previous studies demonstrating the expression of α_6_GABA_A_ receptors mediating an inhibitory tonic current in cerebellar granule cells, relevant to the control of their excitability and therefore fundamental for the function of the cerebellum [1], [8], [22].

### Furosemide-sensitive GABA_A_ receptors modulate motoneuron excitability

Previously we showed that motoneuron excitability might be modulated by high affinity GABA_A_ receptors mediating a tonic inhibitory current [14], [16], and that the monosynaptic reflex is facilitated by furosemide without affecting the dorsal root potential [15]. Taken together, these results suggested the presence of furosemide-sensitive GABA_A_ receptors in motoneurons. Here, we found that the R_in_ and the excitability of motoneurons recorded intracellularly from thick slices of the turtle spinal cord were increased in the presence of furosemide. This observation is in agreement also with the results observed in the cerebellar granule cells [8], [25]. Therefore, our results suggest that the monosynaptic reflex facilitation we observed in the presence of furosemide [15], might be due to the blockade of furosemide-sensitive GABA_A_ receptors activated by endogenous GABA that shunt the motoneuron membrane. Interestingly, in some motoneurons furosemide did not affect R_in_ and excitability. This could indicate the presence of motoneurons that do not express furosemide-sensitive GABA_A_ receptors, though the possibility exists that they might express other receptor types containing subunits not sensitive to furosemide, but sensitive to bicuculline and/or picrotoxin [1], [16].

### Motoneurons express a tonic inhibitory current activated by GABA and muscimol

In motoneurons, the fast inward current followed by a steady state recorded in the presence of muscimol and GABA is similar to that observed in cerebellar granule cells [9], [22], [25]. However, in our experiments the activation of the GABA_A_ receptors was evoked at higher concentrations of GABA (>20 µM) which might be attributed to the large myelination of the adult turtle spinal cord that prevents the diffusion of the neurotransmitter. Alternatively, neuronal and/or glial uptake may also contribute to the control of the extracellular GABA concentration. This has been confirmed in cerebellar granule cells where blocking of the GABA transporters produces an increase in the persistent tonic current [21].

Likewise, GABA concentration >30 µM did not produce any fast inward current indicating desensitization of GABA_A_ receptors, though some of them could remain activated mediating a tonic inhibitory current. Therefore, it is conceivable that in this condition the furosemide-sensitive GABAergic tonic current recorded in motoneurons could be mediated by tonic activation of α_6_ subunit-containing GABA_A_ receptors. This result is in agreement with previous reports showing that furosemide (100–300 µM) blocks a GABAergic tonic current mediated by α_6_ subunit-containing GABA_A_ receptors located at extrasynaptic regions [8], [9], [26]. Similarly, in studies with recombinant receptors heterologously expressed in *Xenopus* oocytes, furosemide has been shown to antagonize with high affinity α_6_β_2_γ_2_ (IC_50_, about 10 µM) [7] GABA_A_ receptors. In contrast, the drug seems to block less efficiently GABA_A_ receptors containing α_4_ (IC_50_∼250 µM) [20] and α_1_ subunits (IC_50_>3 mM) [7], [20].

The additional component of the outward current revealed when picrotoxin is added after furosemide has reached its stable effect indicates that other α subunit(s) might be forming part of extrasynaptic GABA_A_ receptors in adult motoneurons. Immunohistochemical and *in situ* hybridization studies point out to the expression of α_2_ and α_5_ subunits in rat motoneurons [10], [23], [27]. In line with this, we have shown that extrasynaptic α_5_GABA_A_ receptors sensitive to L-655,708 mediate tonic inhibition of interneurons in the ventral horn of the spinal cord as well as in primary afferents [17], [28]. Therefore, this subunit could be a good candidate to form extrasynaptic GABA_A_ receptors expressed in motoneurons. Interestingly, furosemide did not affect the tonic current in ventral horn interneurons [28] suggesting that at this concentration the Cl^−^ transporter is not affected [29], and supporting our conclusion that the drug is blocking a tonic GABAeregic current mediated by α_6_-containing GABA_A_ receptors in motoneurons. In addition, our molecular biology and immunohistochemical data showed the expression of the α_6_ subunit in the spinal cord, and in particular in motoneurons. To the best of our knowledge, this is the first study reporting the localization and functional expression of the α_6_ subunit in the spinal cord.

### Expression of α_6_ in motoneurons

With the exception of α_6_, the mRNAs encoding all α subunits has been reported in rat motoneurons [11], [12], [23], although the α_4_ subunit mRNA has been reported only in motoneurons of a transgenic mouse overexpressing a mutated variant of the gene coding for the superoxide dismutase 1, which is an animal model that clinically resemble amyotrophic lateral sclerosis (ALS) [13]. Therefore, the current report is the first showing that the mRNA for the α_6_ subunit of the GABA_A_ receptor is expressed in motoneurons. Consistent with this, immunohistochemical analysis showed the presence of the α_6_ subunit in identified motoneurons. Together with our functional and pharmacological analysis, our results support the conclusion that the furosemide-sensitive tonic current recorded in motoneurons may be mediated by α_6_ subunit-containing GABA_A_ receptors.

### Functional implications

Extrasynaptic GABA_A_ receptors expressed in motoneurons and activated by ambient GABA might have important roles in producing a tonic shunt that may decrease excitability and prevent anomalous activation of APs. This role has been suggested in the activation of the monosynaptic reflex [15]. In the presence of furosemide, the synchronous compound AP evoked by one electrical shock applied to the primary afferents is followed by a long lasting discharge of APs. Given that the drug does not affect the Cl^−^ transporter at the concentration used, it is reasonable to speculate that the long lasting component after motoneuron discharge in our experiments may be associated to an effect on the inhibitory current mediated by α_6_GABA_A_ receptors. Therefore, it can be suggested that these receptors may play a role in motor control.
